# Caffeine Augments The Instruction of Anti-Inflammatory Macrophages by The Conditioned Medium of Mesenchymal Stem Cells 

**DOI:** 10.22074/cellj.2017.4364

**Published:** 2017-08-19

**Authors:** Nazanin Shushtari, Seyyed Meysam Abtahi Froushani

**Affiliations:** Division of Immunology, Department of Microbiology, Faculty of Veterinary Medicine, Urmia University, Urmia, Iran

**Keywords:** Mesenchymal Stem cell, Conditioned Medium, Macrophage, Caffeine

## Abstract

**Objective:**

Mesenchymal stem cells (MSCs) have been shown to produce adenosine, express adenosine receptors, and communicate with macrophages and other cells. However, there is no information about the role of caffeine, as a popular drink and adenosine antagonist, on the crosstalk between MSCs and immune cells. The aim of the current study is to evaluate the effects of the conditioned medium of MSCs treated with caffeine on macrophages.

**Materials and Methods:**

In this experimental study, MSCs were isolated from bone marrow of rats and pulsed with different concentrations of caffeine (0, 0.1, 0.5 and 1 mM) for 72 hours. The conditioned medium of MSCs was collected after 24 hours, then incubated with macrophages for 24 hours. Finally, the functions of the macrophages were evaluated.

**Results:**

Conditioned medium of MSCs treated with caffeine significantly enhanced phagocytosis and simultaneously regressed expression of reactive oxygen species (ROS) and nitric oxide (NO) as well as IL-12 by macrophages compared to the supernatants of MSCs alone. The conditioned medium of MSCs pulsed with caffeine at low to moderate concentrations preserved the neutral red uptake by macrophages and elevated IL-10 secretion by macrophages. A high concentration of caffeine could interfere with the two latter effects of supernatants of MSCs on the macrophages.

**Conclusion:**

Collectively, caffeine treatment of MSCs appeared to augment the instruction of anti-inflammatory macrophages by conditioned medium of MSCs. These findings might offer new insight into the potential mechanisms that underlie the immunomodulatory and anti-inflammatory effects of caffeine.

## Introduction

Mesenchymal stem cells (MSCs) are a population of self-renewable multipotent progenitor cells that have the potential to give rise to cells of various mesenchymal lineages that include fat, cartilage, and bone (1). They are generally found in the bone marrow and numerous other tissues such as adipose tissue, umbilical cord blood (2), dermal tissue (3), and peri-endothelial areas (4). The scientific literature suggests that MSCs possess potent immunoregulatory properties (5,6). Preliminary and clinical studies along with multiple animal models indicate that MSC therapy is a worthwhile strategy to down-regulate pathogenic immune responses in graft-versus-host and autoimmune diseases (7). 

Caffeine (1,3,7-trimethylxanthine) is a natural xanthine alkaloid found in coffee, tea, soft drinks, chocolate, kola nuts, and certain medicines (8, 9). Beverages that contain caffeine are commonly consumed worldwide, especially in Europe and North America (10). Since caffeine is both water- and lipidsoluble, it is readily distributed throughout different tissues (11). Caffeine diversely influences various body systems such as the endocrine, cardiovascular, respiratory, urinary, gastrointestinal metabolism, immune, and especially the central nervous system (12, 13). Caffeine is the world’s most widely and legally consumed psychoactive agent (12). Structurally, caffeine is similar to adenosine and acts as a competitive inhibitor (14, 15). Recent works have presented that MSCs produce adenosine and express adenosine receptors (A1R, A2AR, A2BR, and A3R). Therefore, stimulation of these receptors may play an active role in mouse bone marrow-derived MSC proliferation and differentiation (16).

MSCs in the tissue and bone marrow niche provide an environment that has an inevitable crosstalk with hematopoietic cells including macrophages (17-19). Although the interactions of MSCs with numerous cells of the immune system have been investigated in researches (1, 19, 20), very little is known about the interaction between MSCs and macrophages. On the other hand, there is no information about the role of caffeine as a popular environmental factor in the crosstalk between MSCs and immune cells. The present investigation has sought to assess the effects of the conditioned medium of MSCs treated with caffeine on macrophage functions.

## Materials and Methods

In this experimental study, we obtained tetradecanoylphorbol acetate (TPA), lipopolysaccharide (LPS), caffeine, natural red (NR), nitroblue tetrazolium (NBT), dioxin, dimethyl sulfoxide (DMSO), 3-[4,5-dimethylthiazol-2-yl]-2,5-diphenyltetrazolium bromide (MTT), phytohemagglutinin (PHA) and phosphate-buffered saline (PBS) from Sigma-Aldrich (St. Louis, MO). In addition, fetal calf serum and Dulbecco’s Modified Eagle’s Medium (DMEM) were purchased from Gibco/Life Technologies, Inc. (Gaithersburg, MD). The monoclonal antibodies used in flow cytometric analysis were purchased from Becton Dickinson.

Male Wistar rats (8-10 weeks old) were obtained from the Experimental Animal Center of the Veterinary Faculty of Urmia University, Urmia, Iran. The rats were maintained under constant temperature (22-24˚C) with a 12-hour light/dark cycle, and received food and water ad libitum. Animal welfare was conducted in compliance with regulations of the Ministry of Health and Medical Education of the I.R. Iran, and approved by the Medical Ethics Committee of the University for Animal Studies.

### Isolation, proliferation, and characterization of mesenchymal stem cells

Bone marrow derived MSCs were obtained on the basis of their ability to adhere to the culture plates as previously described (21). In brief, the bone marrow cells from deeply anesthetized Wistar rats were collected by flushing the femurs and tibias, then washed twice by centrifugation at 1200 rpm for 5 minutes in PBS. The isolated cells were plated in 75-cm^2^ tissue culture flasks at concentrations of 0.3 to 0.4×10^6^ cells/cm^2^ in DMEM medium, supplemented with 15% heatinactivated fetal calf serum. The cells were incubated in a humidified 5% CO_2_ incubator at 37˚C. On the third day, we removed any non-adherent cells and added fresh medium for further growth. These cultures were fed twice per week and upon 70% confluency, the cells were removed using trypsin/EDTA, then counted and passed at a 1:3 ratio (approximately 1.5×10^6^ cells/75-cm^2^ flask). The cell passage was performed up to sub-culture 3.

Afterwards, MSCs were immunophenotyped by flow cytometry as previously described (22). Briefly, cells (5×10^5^ in 100 μl PBS/0.5% bovine serum albumin (BSA) and 2 mmol ethylenediaminetetraacetic acid (EDTA) were mixed with 10 μl of the fluorescent labeled monoclonal antibody [anti-rat CD29 (Integrin b chain; Ha2/5; FITC), CD45-FITC and CD90- PCY5 (Thy-1/Thy-1.1-FITC)] and incubated in the dark at 2-8˚C for 30 minutes. The stained cells were rinsed twice with PBS that contained 2% BSA and the pellet was re-suspended in PBS. Cell fluorescence was analyzed immediately on a DAKO flow cytometer (Partec, Germany).

### Lymphocyte proliferation assay

In brief, spleens were aseptically isolated from 3 rats. Next, we prepared single-cell suspensions of splenocytes as described earlier. The splenocytes were cultured in 96-well flatbottomed plates (1×10^5^ cells/100 μl/well) in and stimulated with PHA (final concentration of 5 μg/ ml) or medium alone. Plates were also cultured in the presence (10 splenocytes to 1 MSC) or absence of MSCs. After 5 days, the cells were pulsed with 20 μl of the MTT solution (final concentration: 5 mg/ml) for 4 hours at 37˚C. Next, to dissolve the formazan crystal, 150 ml DMSO was mixed and shaken vigorously. The optical density (OD) at 550 nm was determined by a microplate reader (Dynatech, Denkendorf, Germany). The experiments were performed in triplicate. The results were expressed as the proliferation index (PI) on the MTT assay calculated according to the ratio of OD550 of stimulated cells with PHA to OD550 of nonstimulated cells (23).

### Treatment of mesenchymal stem cells with caffeine and collection of supernatants

We incubated the passage-3 MSCs with different concentrations of caffeine (0.1, 0.5, and 1 mM) at different times (24, 48, and 72 hours). After aspiration of the medium, we washed the cells three times with PBS. Then, the cells were cultured with serum-free DMEM for 24 hours. The supernatants were collected and centrifuged for 10 minutes at 300 g and filtered through a 0.2-μm filter to remove the cellular debris.

### Peritoneal macrophage isolation

The resident macrophages were collected from the peritoneal cavity of Wistar rats by injecting 20 ml of ice-cold PBS. The peritoneal fluid was withdrawn and centrifuged at 600 g for 10 minutes at 4˚C. The pellets were washed twice in PBS and suspended in DMEM that contained 10% heat-inactivated FCS. The cells were counted in Neubauer chamber and we assessed their viability by trypan blue dye exclusion. Then, 100 μl of the live cell suspension (2×10^6^ cells/ml) was pre-incubated in 96-well microplates for 40 minutes at 37˚C in a moist atmosphere of 5% CO_2_. This method supported macrophage adherence to the plate. The non-adherent cells were discarded by vigorously washing three times with icecold PBS. Differential counts of the adherent cells used for the experiments were assessed microscopically after Giemsa staining. The cell viability examined by trypan blue exclusion was never below 96%.

### Macrophage incubation with mesenchymal stem cell-conditioned medium

We added 25 μl of the collected conditioned medium to each well of 96-well microplates that contained macrophages, and then incubated the microplates for 24 hours at 37˚C in a moist atmosphere of 5% CO_2_. At the end of the incubation period, the media were removed and replaced by fresh media.

### Neutral red uptake

After co-culture of the conditioned medium of MSCs with macrophages, 200 μl neutral red solution (dissolved in 10 mmol/L PBS with a concentration of 0.075%) was added and incubated for 1 hour. The supernatant was eliminated and the cells in 96-well plates were washed twice with PBS to remove the neutral red that was not phagocytized by macrophages. Then, the lysing buffer (ethanol and 0.01% acetic acid at a 1:1 ratio, 200 μl/well) was added to lyse the cells. When the cells were incubated overnight at 4˚C, the OD at 490 nm was measured by a microplate reader (Dynatech, Denkendorf, Germany).

### Macrophage phagocytosis

Macrophages were incubated with neutralred-stained, heat-stabilized, zymosan suspension for 30 minutes at a 1:10 ratio as previously described (24). We removed the supernatant and phagocytosis was stopped by the addition of Baker’s formol calcium solution. The cells were washed 3 times by centrifugation in PBS. Neutral red was solubilized by acidified alcohol and the absorbance was evaluated spectrophotometrically at 550 nm.

### Respiratory burst of activated macrophages

The respiratory burst of activated macrophages was evaluated as previously explained with some modifications (25,26). The macrophages were incubated for 20 minutes with 100 ng/ml TPA and 0.1% NBT. The unexploited NBT dye was deleted through washing and the reduced dye was extracted in dioxin and measured at 520 nm. 

### Macrophage nitric oxide production

The isolated macrophages were stimulated with LPS (10 pg/mL) for 24 hours. After this period, the potential for nitric oxide (NO) production was determined by the Griess method. The cell-free supernatants (50 µl) were collected and mixed with 50 µl Griess reagent (0.1% sulfanilamide, 3% phosphoric acid, and 0.1% naphthyl ethylenediamine), then allowed to incubate at room temperature for 10 minutes in the dark. After incubation, the absorbance was quantitated at 540 nm on a microplate reader (Dynatech, Denkendorf, Germany). The nitrite concentration was estimated based on the standard curve. 

### Cytokine assay

The macrophages were stimulated with LPS (10 pg/mL) for 24 hours. We collected the cell- free supernatant and assayed IL-10 and IL-12 levels using an ELISA kit (Bender MedSystems, Austria) according to the manufacturer’s instructions. 

### Statistical analysis

The normal distribution of data was confirmed with the Kolmogorov-Smirnov test. Next, the data were analyzed using one-way ANOVA plus Dunnett’s post-hoc test, and subsequently presented as means ± SD. P<0.05 were considered statistically significant. 

## Results

**Fig.1:**
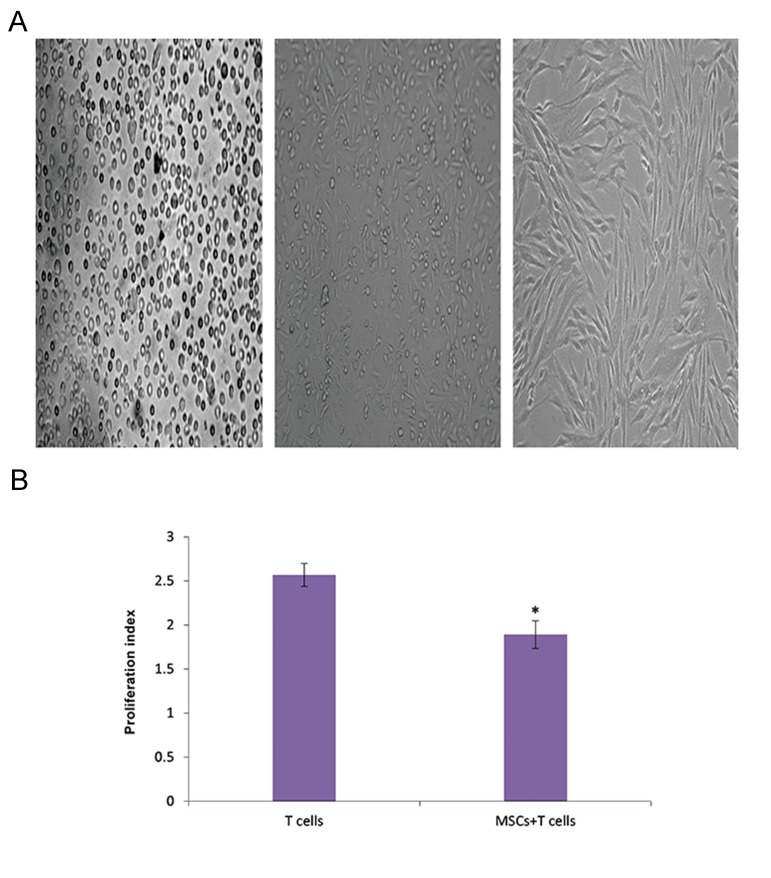
Characterization of mesenchymal stem cells (MSCs). A. Representative fields depicted rat bone marrow-derived MSCs at different passages. Left: Initially, the isolated cells showed a round morphology. Middle: During the first sub-culture, MSCs exhibited diverse morphologies that included ovoid, bipolar and large, flattened morphology. Right: Sub-culture 3 of cells showed large, flattened or fibroblast-like morphology typical of MSCs and B. MSC-T cell co-culture: T cells were stimulated with phytohemagglutinin (PHA) or medium in the presence of MSCs (10 splenocytes to 1 MSC) or absence of either MSCs or MCs. After 5 days, we calculated the proliferation index (PI). *; P<0.01 vs. T lymphocytes without MSCs.

Adherent cells isolated from bone marrow gradually grew into small colonies. The sub- culture 3 adherent cells showed a homogeneous fibroblast-like, spindle-shaped morphology typical of MSCs (Fig .1A). Inhibition of the poly-clonal T lymphocyte proliferation is a hallmark of bone marrow MSCs (27). We found that isolated MSCs also inhibited proliferation of polyclonally stimulated T lymphocytes (Fig .1B). Flow cytometric data demonstrated that the sub-culture 3 isolated cells tested positive for MSC markers of rats (CD29 and CD90), but were negative for the marker for hematopoietic cells (CD 45, Table 1). 

**Table 1:** The immunophenotypic characterization of isolated mesenchymal stem cells


Marker	Sample 1%	Sample 2%	Sample 3%	Mean ± SD%

CD29	98.29	96.34	98.54	97.41 ± 0.98
CD45	2.14	2.89	1.82	2.28 ± 0.54
CD90	95.76	94.36	91.87	93.99 ± 1.97


**Fig.2:**
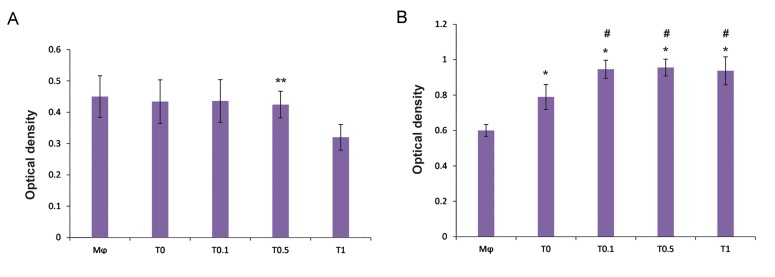
Evaluation of phagocytic potential of macrophages. A. Modulation of neutral red uptake and B. Phagocytosis of neutral red stained zymosan by macrophages (Mφ). Macrophages co-cultured with the conditioned medium of mesenchymal stem cells (MSCs) pulsed with different concentrations of caffeine [0.1 (T0.1), 0.5 (T0.5), and 1 (T1) mM] or the conditioned medium of MSCs alone (T0) for 24 hours. **; P<0.001 vs. other groups, *; P<0.001 vs. Mφ, and #; P<0.001 vs. T0.

The NR uptake by macrophages did not exhibit any significant difference between macrophages alone, macrophages co-cultured with the conditioned medium of MSCs alone, or with the conditioned medium of MSCs treated with caffeine at concentrations of 0.1 and 0.5 mM. However, the conditioned medium of MSCs pulsed with caffeine at a concentration of 1 mM significantly decreased NR uptake by the co-cultured macrophages compared with the other groups (Fig .2A).

The phagocytosis of macrophages significantly increased in the macrophages exposed to the conditioned medium of MSCs alone and the conditioned medium of MSCs pulsed with caffeine compared to macrophages without treatment. However, the increase in phagocytic activity of the co-cultured macrophage was more pronounced in the group exposed to the conditioned medium of MSCs treated with caffeine than the group exposed to the conditioned medium of MSCs alone (Fig .2B). 

**Fig.3:**
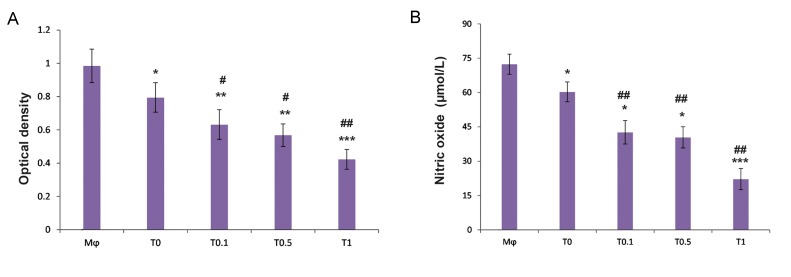
Measurement of oxidative burst and nitric oxide production in macrophages. A. Evaluation of macrophage (Mφ) respiratory burst after activation by tetradecanoylphorbol acetate (TPA) and B. Nitric oxide (NO) production of macrophages after challenge with lipopolysaccharide (LPS). Mesenchymal stem cells (MSCs) were pulsed with different concentrations of caffeine [0.1 (T0.1), 0.5 (T0.5), and 1 (T1) mM] for 72 hours. Then, MSCs were cultured for 24 hours without treatment after which we collected the conditioned medium of MSCs. Macrophages were incubated for 24 hours with the conditioned medium of MSCs pulsed with caffeine or the conditioned medium of MSCs alone (T0). The respiratory burst and NO production of macrophages were assessed by thenitroblue tetrazolium (NBT) reduction assay and Griess method, respectively. *; P<0.05, **; P<0.01, ***; P<0.001 vs. Mφ, #; P<0.01, and ##; P<0.001 vs. T0.

**Fig.4:**
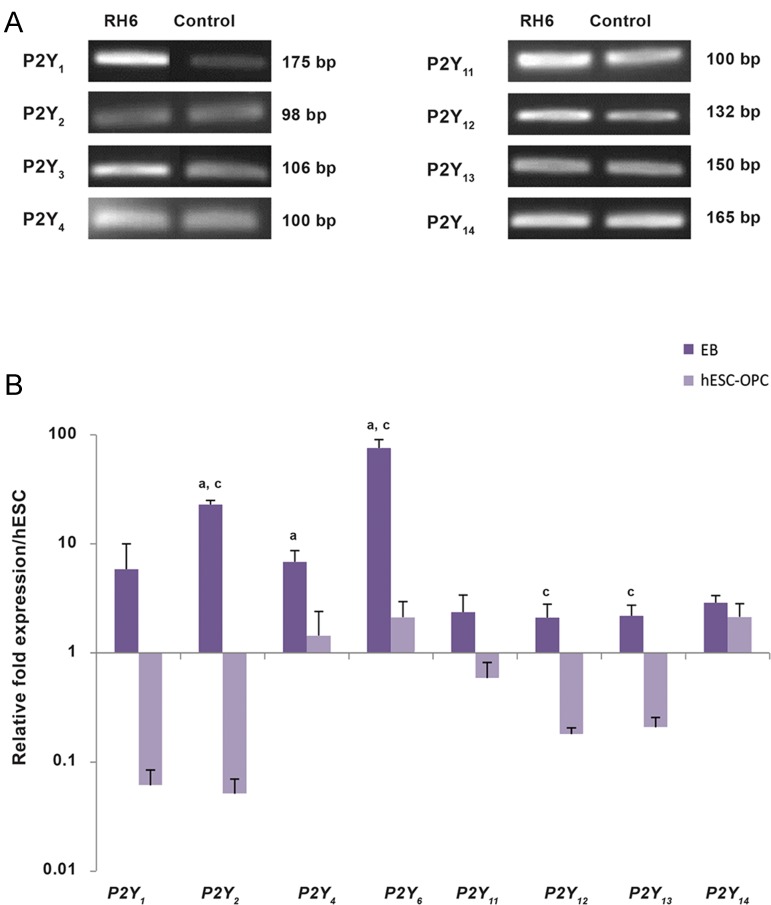
Cytokine modulation by conditioned medium of mesenchymal stem cells (MSCs). A. IL-12 production by macrophages (Mφ) after challenge with conditioned medium of MSCs and B. The effect of conditioned medium of MSCs on IL-10 production by macrophages. *; P<0. 001 vs. Mφ, #; P<0.05, ##; P<0.01, and ###; P<0.001 vs. T0.

The obtained findings showed that the conditioned medium of MSCs could meaningfully down- regulate respiratory burst and NO production by macrophages compared with macrophages alone (Fig .3A). In addition, the conditioned medium of MSCs pulsed with caffeine considerably lowered the respiratory burst and NO production of the co-cultured macrophages more than observed in the macrophages co-cultured with the conditioned medium of MSCs alone (Fig .3B). Of note, we observed a significant decrease in IL-12 in the macrophages co-cultured with the conditioned medium of MSCs alone and with the conditioned medium of MSCs treated with caffeine compared to macrophages alone. However, the conditioned medium of MSCs treated with caffeine markedly reduced IL-12 production, which was more prominent than similar findings obtained by the macrophages co-cultured with the conditioned medium of MSCs alone (Fig .4A). The conditioned medium of MSCs significantly up-regulated IL-10 production by macrophages compared to macrophages alone. The conditioned medium of MSCs pulsed with caffeine at low to moderate concentrations notably enhanced IL-10 secretion by the co-cultured macrophages which was greater than documented in the macrophages co- cultured with the conditioned medium of MSCs alone. The conditioned medium of MSCs pulsed with caffeine at high concentrations dramatically decreased IL-10 secretion by the co-cultured macrophages (Fig .4B). 

## Discussion

Adenosine receptor subtypes express in MSCs and can directly produce adenosine. Therefore, adenosine and adenosine receptors possess an autocrine or paracrine role in the function and differentiation of MSCs (28). It has been previously determined that the methylxanthine derivative, like caffeine, can interfere with the biology of adenosine/adenosine receptors (14,15). Although the effects of caffeine at the cellular level have been documented, information on its effect on MSC functions such as their immunoregulatory properties are not fully understood. For the first time, the result of this study has reported that caffeine could alter the immunoregulatory properties of MSCs conditioned medium. 

Neutral red can be ingested and accumulated in the lysosomes of macrophages according to the level of cell activation. Neutral red uptake by macrophages depends on various factors related to cell viability, activity, and cell membrane integrity (29). The obtained data has demonstrated that NR uptake did not show any change between the macrophages alone, macrophages co-cultured with the conditioned medium of MSCs, and macrophages co-cultured with conditioned medium of MSCs pulsed with caffeine at concentrations of 0.1 and 0.5 mm. However, a high concentration of caffeine (1 mM) interfered with the activity state and viability of macrophages, because NR uptake by macrophages decreased after the challenge with the supernatants derived from MSCs treated with caffeine at the 1 mM concentration. Phagocytosis is an essential function of macrophages which takes part in the uptake of pathogens. Additionally, phagocytosis of apoptotic bodies and debris is necessary for tissue resolution and termination of inflammation (30). In addition to the increase in phagocytosis of macrophages cultured with the MSC supernatants, we observed that the conditioned medium of caffeine treated bone marrow-derived MSCs might lead to a significant increase in the phagocytic ability of macrophages compared to MSCs alone. 

Although reactive oxygen species (ROS) and nitrogen species (like NO) participate in host defense, the overproduction of these species may contribute to the pathogenesis of inflammatory and degenerative diseases (31,32). In addition, different stimuli may induce a respiratory burst in macrophages and harm tissues in immunopathologic conditions (17). MSCs could significantly inhibit ROS and NO production by macrophages. The data also showed that the supernatants of the MSCs pulsed with caffeine profoundly suppressed the production of potentially harmful ROS and NO by macrophages more pronounced than MSCs without treatment. A higher phagocytic activity, without producing potentially harmful ROS and NO, could help phagocytes to resolve and terminate inflammation. These findings indicated that the conditioned medium of MSCs pulsed with caffeine at low to moderate doses could exert a protective role from inappropriate, non-specific, and potentially harmful reactions. 

IL-12 is a potent pro-inflammatory cytokine mostly produced by phagocytic cells. IL-12 is one of the main instructors of T cell-dependent inflammation (33). The observations in this research have shown that the production of IL-12 by macrophages co- cultured with conditioned medium of MSCs alone or with the conditioned medium of MSCs treated with caffeine significantly down-regulated compared to macrophages alone. However, this reduction was more prominent in macrophages exposed to the conditioned medium of MSCs treated with caffeine. 

IL-10, a cytokine with anti-inflammatory properties, plays an important function in limiting and terminating inflammatory reactions and subsequently, preventing tissue destruction (34). In this study, it has been observed that secretion of IL-10 by macrophages exposed to the conditioned medium of MSCs alone or to the conditioned medium of MSCs treated with caffeine at low to moderate doses significantly increased compared to macrophages alone. The conditioned medium derived from the MSCs treated with caffeine at high concentrations might eventually become toxic since it caused a dramatic decrease in cytokine production (both IL-10 and IL-12) and NR-uptake. 

Macrophages are substantially plastic and diverse. According to environmental factors, they may undergo reprogramming that can result the in emergence of a variety of different functional phenotypes. (31,32,35). Classically-activated macrophages or M1 macrophages have potent antimicrobial and inflammatory properties, and may contribute to the pathogenesis of inflammatory diseases. Alternatively-activated macrophages or M2 macrophages produce less pro-inflammatory mediators such as NO and ROS, and possess a role in resolution of inflammation via trophic factor secretion and higher phagocytic activity (32,35). Previous works have indicated that macrophages, co-cultured with bone marrow derived MSCs, demonstrated high levels of CD206, a marker of M2 macrophages, and expressed high levels of IL-6 and IL-10 and low levels of IL-12 and TNF-α compared with controls (35). Our results confirmed that similar to MSCs, the conditioned medium of MSCs also educated macrophages towards M2 phenotype via a decrease in the production of NO, ROS, and IL-10 and an increase in phagocytosis and IL-12. As discussed above, low to moderate doses of caffeine may potentiate such effects of the conditioned medium of MSCs. Nonetheless, caffeine at higher doses can eventually become toxic as it decreases the neutral red uptake and cytokine production (both IL-10 and IL-12) by macrophages. 

The dose-dependent effects of caffeine are not unlikely. It has been reported that the direct effects of caffeine on alveolar macrophages confirm a dose-dependent manner: at lower concentrations, caffeine inhibits apoptosis and at higher concentrations, macrophages proceed to apoptosis. The former data have documented that the effects of caffeine on MSCs differentiation are also dose-dependent: a caffeine concentration of 0.1 mM significantly enhanced the osteogenic differentiation and mineralization of rat bone marrow-derived MSCs. Nevertheless, caffeine concentrations greater than 0.3 mM suppressed the osteogenic differentiation of rat bone marrow- derived MSCs (12). 

A previous work indicated that caffeine at concentrations relevant to normal human consumption had anti-inflammatory and immunomodulatory effects (15). Here, in this research, we proposed that some of the immunomodulatory and anti-inflammatory effects of caffeine might be due to the influence of caffeine on the impact of MSCs on macrophages. Autologous cell therapy by M2 macrophages may modulate immune responses and decrease inflammation (36). In this regard, the *in vitro* education of macrophages towards anti- inflammatory phenotype by the conditioned medium of MSCs may possess a therapeutic interest because this method rapidly produces enough autologous cells with anti-inflammatory potentials. Interestingly, the observations in the present research have suggested that caffeine can augment the instruction of anti- inflammatory macrophages by the conditioned medium of MSCs. 

## Conclusion

These data proposed that the conditioned medium of MSCs treated with caffeine could potentiate the education of macrophages toward an anti-inflammatory phenotype through preservation of the activity of macrophages, increased phagocytosis, reduced production of potential harmful ROS and NO by macrophages, and decreased inflammatory cytokine IL-12 which was more profound than the conditioned medium of MSCs alone. The conditioned medium of MSCs pulsed with caffeine at low to moderate concentrations preserved neutral red uptake and elevated anti-inflammatory IL-10 by macrophages. High concentrations of caffeine could interfere with the two latter effects of the supernatants of MSCs on macrophages and eventually become toxic. Collectively, the present findings could offer a new insight into the potential mechanisms that underlie the immunomodulatory and anti- inflammatory effects of caffeine. 
